# Clonal diversity of the B cell receptor repertoire in patients with coronary in-stent restenosis and type 2 diabetes

**DOI:** 10.1515/biol-2021-0091

**Published:** 2021-08-28

**Authors:** Ruiqiang Weng, Sudong Liu, Xiaodong Gu, Zhixiong Zhong

**Affiliations:** Research Experimental Center, Meizhou People’s Hospital (Huangtang Hospital), Meizhou Hospital Affiliated to Sun Yat-Sen University, Meizhou 514031, People’s Republic of China; Guangdong Provincial Engineering and Technological Research Center for Molecular Diagnostics of Cardiovascular Diseases, Meizhou 514031, People’s Republic of China; Provincial Key Laboratory of Precision Medicine and Clinical Translational Research of Hakka Population, Meizhou 514031, People’s Republic of China; Center for Precision Medicine, Meizhou People’s Hospital (Huangtang Hospital), Meizhou Hospital Affiliated to Sun Yat-sen University, Meizhou 514031, People’s Republic of China; Center for Cardiovascular Diseases, Meizhou People’s Hospital (Huangtang Hospital), Meizhou Hospital Affiliated to Sun Yat-sen University, Meizhou 514031, People’s Republic of China

**Keywords:** B cell receptor repertoire, repertoire sequencing, in-stent restenosis, type 2 diabetes mellitus

## Abstract

Type 2 diabetes mellitus (T2DM) is known as a risk factor for coronary in-stent restenosis (ISR) in patients with coronary artery disease (CAD). Evidence suggests that B cells play a functional role in the progression of atherosclerotic lesions. However, the B cell receptor (BCR) repertoire in patients with ISR remains unclear. This study aims to profile the BCR repertoire in patients with coronary ISR/T2DM. A total of 21 CAD patients with or without ISR/T2DM were enrolled. PBMCs were isolated and examined for BCR repertoire profiles using DNA-seq. Our results showed that the diversity of amino acid sequences in ISR DM patients was higher than that in ISR −DM patients. The frequencies of 21 V/J paired genes differed between ISR DM and −ISR DM patients, while frequencies of 5 V/J paired genes differed between ISR DM and ISR −DM. The −ISR −DM group presented the highest clonotype overlap rate, while ISR DM patients presented the lowest overlap rate. Our study presented the BCR repertoires in patients with ISR/T2DM. The data suggested different BCR signatures between patients with ISR and T2DM. Further analysis of BCR profiles would enhance understanding of ISR.

## 1 Introduction

Coronary artery disease (CAD) is caused by atherosclerosis, defined as proliferation, hypertrophy, and calcareous deposition in the arterial wall, resulting in reduced vascular elasticity, thrombosis, occlusion, stenosis, and other changes [1]. Coronary stent implantation has enabled management of the early complications associated with plain balloon angioplasty. A stent implantation is currently the main percutaneous coronary intervention (PCI) and has great advantages over balloon angioplasty [2]. Stent implantation can decrease the frequency of restenosis by preventing elastic recoil and constrictive remodeling. In particular, drug-eluting stent (DES) implantation has led to a 5–10% reduction in the occurrence of in-stent restenosis (ISR) [3]. The DES was coated with antibiotics and immune agents that inhibit intimal hyperplasia on its outer layer, allowing the drug to slowly penetrate into the blood vessels and avoid blood vessel obstruction by scar tissue formed after implantation. ISR is currently the major cause for recurrence of exertional angina pectoris or acute coronary syndromes after coronary angioplasty. Despite the lower occurrence of ISR through advances in stent design and polymers, it is challenging to treat stenosis once it has occurred in these stents.

Studies have enhanced our understanding and awareness of various factors that can increase the risk of clinical and angiographic restenosis [4,5]. Among these risk factors, the most important is diabetes mellitus (DM) [1,6]. The underlying mechanism for the higher incidence of ISR in DM patients is likely to be complex because the B cells and T cells involved in balancing the immune state contribute to ISR. Recent studies demonstrated that B cells have a pro-inflammatory role in inflammatory diseases like CAD [7,8,9]. B2 cells produce tumor necrosis factor (TNF)-α and IL-10, which act as proatherogenic cytokines [10,11]. The cytokines produced by B cells enhanced immunomodulation during chronic inflammation [12]. Furthermore, the inflammation contributed to plaque formation and modulated the clinical outcomes for thrombotic complications of atherosclerosis(9). Depletion of mature B2 cells by anti-BAFF antibodies resulted in proatherogenic chemokine production by macrophages [13]. B cell depletion reduced the development of atherosclerosis and was also a promising therapy for DM [10,14]. However, B cell-deficient mice failed to resolve experimental autoimmune encephalomyelitis depending on strict B cell-derived IL-10 production [15]. These findings indicated that DM exaggerated B cell function in atherosclerosis.

B cell produces specific antibodies in response to antigens and plays a vital role in protecting the body. B cell receptor (BCR) is composed of immunoglobulin heavy chains (IgH) and immunoglobulin light chains (IgL). IgH are encoded by recombined VDJ genes developed from Variable (V), Diversity (D), and Joining (J) genes (IGHV, IGHJ, IGHD), while IgL are encoded by VJ rearrangements of V genes and J genes. The diversity of BCR was decided by the complementarity-determining region 3 (CDR3), the region that binds antigens [16]. In the present study, we investigated the clonal diversity of the BCR repertoire in coronary ISR patients with DM to provide a reference for BCR sequences in future investigations.

## 2 Materials and methods

### 2.1 Patients and study design

From January 2017 to December 2018, 21 patients with or without ISR and DM at the Center for Cardiovascular Diseases, Meizhou People’s Hospital, were enrolled in the study. The DM patients had been diagnosed with DM for more than 3 years, and all had been diagnosed with CAD, undergone PCI, and been examined for ISR within 1 year. Any lesions were confirmed by coronary CT angiography. The patients were divided into four groups: ISR with DM (ISR DM; *n* = 6), ISR without DM (ISR −DM, *n* = 5), DM without ISR (−ISR DM, *n* = 5), without ISR and DM (−ISR −DM, *n* = 5).

**Informed consent:** Informed consent has been obtained from all individuals included in this study.**Ethical approval:** The research related to human use has been complied with all the relevant national regulations, institutional policies, and in accordance with the tenets of the Helsinki Declaration and has been approved by the Ethics Committee of Meizhou People’s Hospital (Huangtang Hospital), Meizhou Hospital Affiliated to Sun Yat-sen University, Guangdong, China.

### 2.2 Clinical characteristics of the patients

Peripheral blood samples were obtained from the four groups of CAD patients using EDTA anticoagulant tubes. The clinical characteristics were collected through a detailed medical history, physical examination with vital signs, and blood analyses. Exclusion criteria were autoimmune diseases or neoplasms, treatment with corticosteroids or other immunomodulatory therapy, or vaccination within 3 months before or after PCI. All parameters were measured in the Clinical Laboratory at Meizhou People’s Hospital using standard protocols.

### 2.3 Sample collection and repertoire sequencing

Whole blood samples (10 mL) were collected from the patients and the peripheral blood mononuclear cells (PBMCs) were immediately isolated with Ficoll-Paque (GE Healthcare, Boston, USA) according to the manufacturer’s instructions. Genomic DNA was extracted from the PBMCs with a PureLink Pro 96 Genomic DNA Purification Kit (Invitrogen, California, USA) and used as a template for multiplex PCR with a Multiplex PCR Kit (Qiagen, Dusseldorf, Germany). The details of the PCR primers are shown in Table 1. The Multiplex PCR protocol was as follows: pre-denaturation at 95°C for 15 min; 30 cycles at 94°C for 30 s, 60°C for 90 s, and 72°C for 30 s; final extension at 72°C for 5 min. Multiplex PCR amplification was done to construct the sequence library and the library was test to qualified for machine sequencing. High-throughput sequencing of each captured library ensured that the sequencing data volumes met the requirements. The amplicons were gel-extracted and purified prior to library preparation and the samples were sequenced as 150-bp paired-end runs on a HiSeq™ Xten machine (Illumina, California, USA).

**Table 1:** The primers for multiplex PCR

Primers	Sequence
IGHV1-18	AGAGTCACCATGACCACAGAC
IGHV1-2/1-46	AGAGTCACCAKKACCAGGGAC
IGHV1-24	AGAGTCACCATGACCGAGGAC
IGHV1-3/1-45	AGAGTCACCATTACYAGGGAC
IGHV1-69/1-f	AGAGTCACGATWACCRCGGAC
IGHV1-8	AGAGTCACCATGACCAGGAAC
IGH2-70/26/5	ACCAGGCTCACCATYWCCAAGG
IGHV3	GGCCGATTCACCATCTCMAG
IGH4	CGAGTCACCATRTCMGTAGAC
IGHV5-51	CAGCCGACAAGTCCATCAGC
IGHV6-1	AGTCGAATAACCATCAACCCAG
IGHV7	GACGGTTTGTCTTCTCCTTG
IGHJ	CTGAGGAGACGGTGACCRKKGT

PCR: polymerase chain reaction (PCR); IGHV: immunoglobulin heavy chain variable region; IGHJ: immunoglobulin heavy joining.

### 2.4 BCR sequence analysis

The bioinformatics analysis was performed as follows: (1) quality control of raw data: Phred quality of >30 in at least 80% and error rate of <0.1%; (2) data filtering: Trimmatic was used to filter out adaptor and barcode sequences, and Flash was used for overlapping of the reads [17]; (3) alignment blast: without mismatches and indel paired-end and single end-reads, the merged paired reads were confirmed identical by MiXCR [18] and aligned to the V, D, and J gene reference sequences in the IMGT database (http://www.imgt.org/) as previously described [19]; (4) characteristic gene-specific sequences (such as CDR3) were extracted from the aligned clone sequences. High-quality V, D, and J gene clone sequences were spliced into BCR clones for further analysis.

### 2.5 Statistical analysis and graphing

Statistical analyses were performed using SPSS 19 with GraphPad Prism software for graphing. All data were presented as mean ± SD, and comparisons between groups were performed by one-way ANOVA. Categorical variables were expressed as frequency and compared using Chi-square (*x* ^2^) test or Fisher’s exact test. The diversity of the BCR repertoire was calculated by the Simpson index, Chao 1 index, and Shannon–Wiener index. Values of *P* < 0.05 were considered statistically significant. The BCR overlap was calculated as previously described [20], based on the number of common amino acid clonotypes in two samples as follows: (number of common amino acid clonotypes in two samples × 2)/(total number of amino acid clonotypes in sample 1 + total number of amino acid clonotypes in sample 2) × 100. The average of all samples in each group was reported.

## 3 Results

### 3.1 Clinical characteristics of patients with ISR and DM

Patients who had undergone PCI with or without ISR and DM were enrolled in the study. Peripheral blood samples were collected from the patients and sent to the Clinical Laboratory for analysis. The clinical and laboratory characteristics of the patients are summarized in Table 2. In addition, we collected risk factor, medication, biochemical, and hematological variables data and performed comparisons among the groups. Significant differences in lipid metabolism and hematological variables like white blood cells and neutrophils were observed (Table 2), indicating that ISR and DM can trigger immune state changes.

**Table 2:** Characteristics of study population at baseline

	ISR DM	−ISR DM	ISR −DM	−ISR −DM	*P*-value^#^
Age ± SD	60.2 ± 7.28	63.8 ± 9.0	63 ± 5.41	61.4 ± 6.82	n.s.
Male/female	3M/3F	3M/2F	2M/3F	3M/2F	n.s.
Risk factor					
Smoking status, *n* (%)	2 (33%)	1 (20%)	2 (40%)	4 (80%)	n.s.
Hypertension, *n* (%)	3 (50%)	3 (60%)	1 (20%)	0	n.s.
Diabetes mellitus, *n* (%)	5 (83%)	5( 100%)	0	0	NA
Medication					
β-blockers, *n* (%)	2 (33%)	3 (60%)	3 (60%)	4 (80%)	n.s.
Aspirin, *n* (%)	3 (50%)	2 (40%)	4 (80%)	4 (80%)	n.s.
ACE inhibitors, *n* (%)	2 (33%)	2 (40%)	1 (20%)	4 (80%)	n.s.
Clopidogrel, *n* (%)	3 (50%)	3 (60%)	1 (20%)	4 (80%)	n.s.
Statins, *n* (%)	6 (100%)	6 (100%)	6 (100%)	6 (100%)	n.s.
Biochemistry variables					
Total Cholesterol (mmol/L)	4.00 ± 1.34	5.00 ± 0.47	4.54 ± 0.69	3.91 ± 0.57	<0.01
LDL (mmol/L)	2.16 ± 1.12	2.71 ± 0.76	2.21 ± 0.69	2.14 ± 0.49	n.s.
HDL (mmol/L)	1.30 ± 0.38	0.93 ± 0.10	1.68 ± 0.29	1.13 ± 0.29	<0.01
Triglycerides (mmol/L)	1.32 ± 0.41	3.74 ± 2.42	1.17 ± 0.32	1.73 ± 0.73	<0.05
Apolipoprotein A1 (g/L)	1.08 ± 0.21	0.87 ± 0.16	1.33 ± 0.12	0.95 ± 0.11	<0.01
Apolipoprotein B (g/L)	0.64 ± 0.24	0.83 ± 0.22	0.65 ± 0.13	0.70 ± 0.15	n.s.
Homocysteine (µmol/L)	15.2 ± 2.20	16.14 ± 6.23	13.5 ± 2.02	17.6 ± 4.0	n.s.
Baseline glucose (mmol/L)	7.37 ± 1.51	7.64 ± 3.69	5.16 ± 0.71	4.75 ± 0.04	n.s.
HbAc1 (%)	9.1 ± 1.99	7.32 ± 1.33	6.5 ± 0.47	5.92 ± 0.46	n.s.
Hematological variables					
WBCs (10^3^/μL)	7.86 ± 0.41	8.38 ± 1.28	8.96 ± 1.10	6.9 ± 0.59	<0.05
Neutrophil (10^3^/μL)	69.86 ± 6.36	69.2 ± 7.10	70.36 ± 6.83	62.6 ± 8.57	n.s.
Neutrophil (%)	5.5 ± 0.63	5.86 ± 1.39	6.28 ± 0.75	4.34 ± 0.79	<0.05
Lymphocyte (10^3^/μL)	20.16 ± 4.71	24.00 ± 7.34	21.3 ± 5.95	27.8 ± 10.41	n.s.
Lymphocyte (%)	1.66 ± 0.40	1.84 ± 0.29	1.94 ± 0.65	1.92 ± 0.64	n.s.
Monocyte (10^3^/μL)	6.60 ± 0.93	6.00 ± 0.57	7.4 ± 1.59	6.00 ± 1.63	n.s.
Monocyte (%)	0.50 ± 0.08	0.50 ± 0.11	0.64 ± 0.14	0.44 ± 0.13	n.s.

^#^Comparisons between groups were performed using a one-way ANOVA for continuous variables and with a Chi-square test for categorical variables.

WBCs: while blood cells; HbAc1: glycosylated hemoglobin; SD: standard deviation; n.s.: nonsignificant, NA: not applicable.

### 3.2 Different clonal diversity of the BCR repertoire in patients associated with ISR and DM

The study cohort comprised 21 patients with or without ISR and DM. All of the DNA libraries were sequenced and complete BCR repertoire data were successfully obtained using an Illumina HiSeq Xten machine. On average, 5,177,449 (range: 3,839,975–8,001,683) raw Illumina sequencing reads were obtained for each sample. After performing the quality control requirements and data filtering described in the Methods section, an average of 1,799,447 (range: 2,660,587–1,303,608) unique sequence numbers were filtered out for the alignment blast. Detailed descriptions of the sequence numbers are provided in Table A1.

**Figure 1:**
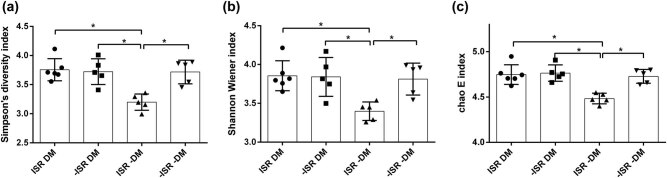
Diversity index of amino acid sequences. (a–c) Calculated diversity index values for amino acid sequences in the four groups. (a) Simpson index values. (b) Shannon–Wiener index values. (c) Chao 1 index values. Each dot represents information for one patient. *P < 0.05, significant difference by one-way ANOVA.

BCR repertoire diversity is a key feature of the humoral immune system and creates the potential for recognition of the wide variety of antigens. To evaluate the BCR repertoire diversity, we first analyzed the Simpson index, Shannon–Wiener index, and Chao 1 index values for amino acid sequences and found that the diversity of amino acid sequences in ISR DM patients was higher than in ISR–DM patients. This meant that DM affected the BCR repertoire diversity in ISR patients (Figure 1). However, in non−DM patients, a decrease in diversity was observed in ISR patients. These results indicated that ISR and DM can both change the diversity of amino acid sequences in patients.

**Figure 2:**
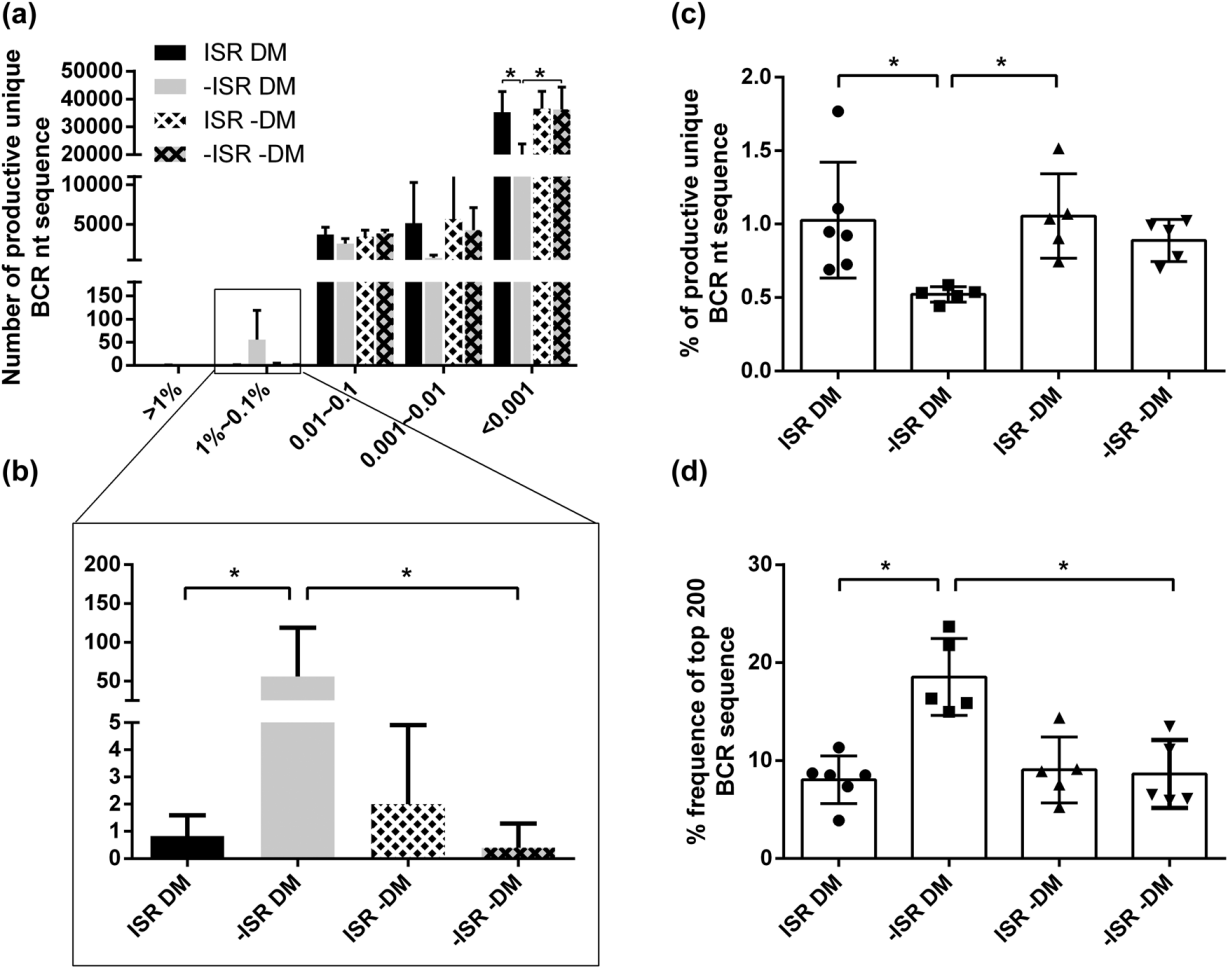
Clonal distribution of BCR repertoires. (a–d) Evaluation of the diversity of BCR repertoires among patients. (a and b) Mean numbers of productive unique BCR nucleotide sequences for five sections (a) and 1–0.1% section in detail (b). The percentages of productive unique BCR nucleotide sequences in the groups are shown. (c) Mean frequencies of productive unique BCR nucleotide sequences among the four groups. (d) Frequencies of the top 200 BCR repertoire nucleotide sequences in the five groups. Data represent the mean distribution ± SD in each group. Each dot represents information for one patient. *P < 0.05, significant difference between groups by a *t*-test. nt: nucleotide.

**Figure A1:**
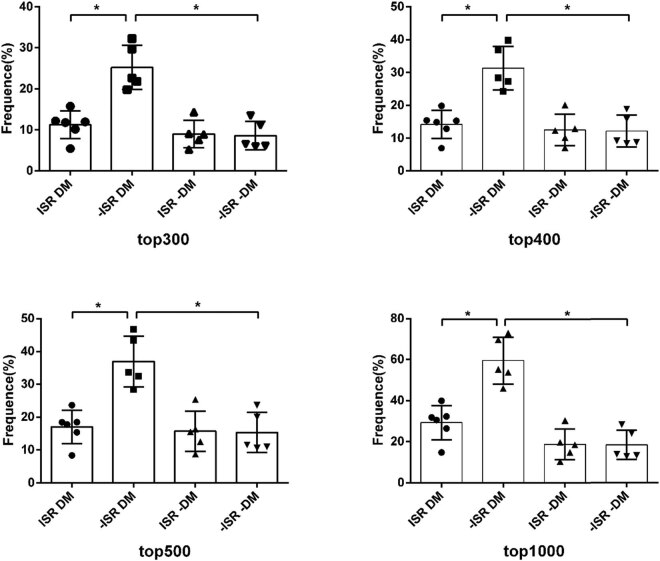
Data show the frequency of top 300, top 400, top 500, top 1,000 B cell receptor repertoire nucleotide sequence in five groups. Groups were compared using Student’s *t*-test. ISR: in-stent restenosis; DM: diabetes mellitus.

To learn more about the diversity, we created five sections based on the frequency of the BCR nucleotide sequences. The results showed that the number of productive unique BCR nucleotide sequences was highest in the 1–0.1% section in −ISR DM patients, but had the lowest frequency in −ISR −DM patients compared with the other groups (Figure 2a and b). Regarding productive unique BCR nucleotide sequences, −ISR DM patients had a lower percentage compared with ISR DM or ISR −DM patients (Figure 2c). Similar results were found in −ISR DM patients for significantly higher percentages of high-frequency nucleotide sequences, regardless of being in the top 200, top 500, or top 1,000 BCR sequences (Figure 2d and Figure A1). These findings suggest that in DM patients, ISR can decrease the low frequency of BCR nucleotide sequence diversity, but increase the number of productive unique BCR nucleotide sequences. These data provide further evidence that ISR and DM can both affect the diversity of amino acid sequences in patients.

### 3.3 Differential V and J gene usage within groups compared with between groups

**Figure 3:**
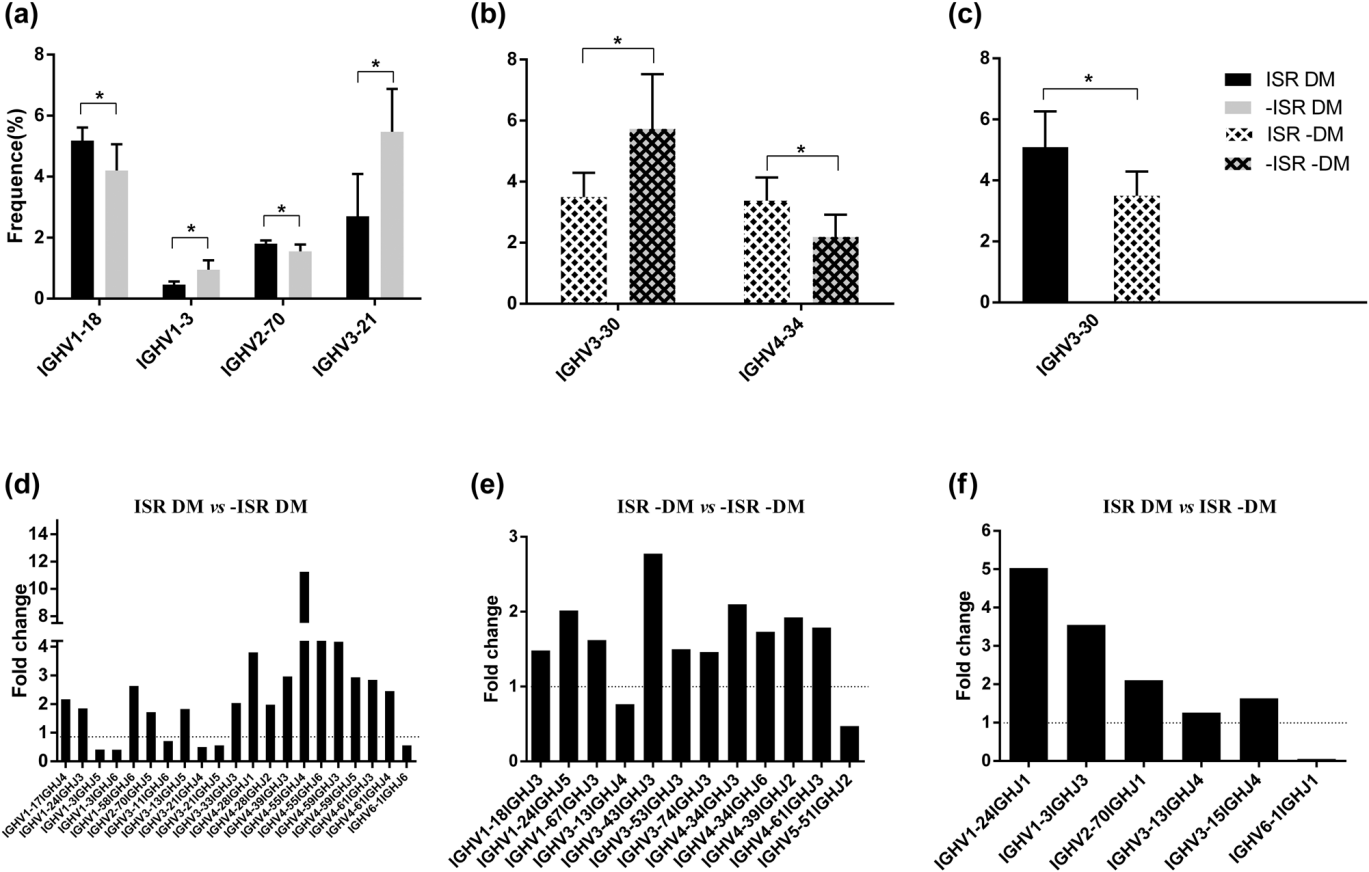
Significant difference of V/J gene usage (a–f). The mean frequency of V subgroup gene usage was shown in each group and the significant difference of V subgroup gene usage for (a) ISR DM compared to −ISR DM, (b) ISR −DM compared to −ISR –DM, and (c) ISR DM compared to −ISR −DM. Data are represented as mean ± SD of the group. Comparison of V/J paired gene usage between groups. The fold change of significant difference usage in V/J paired gene compared between groups for (d) ISR DM compared to −ISR DM, (e) ISR −DM compared to −ISR –DM, and (f) ISR DM compared to ISR −DM was shown. All *P*-values were less than 0.05 by *t*-test. Fold change = the mean frequency in one group divided by the mean frequency in other group.* indicate that the difference is significant (*P < 0.05).

**Figure A2:**
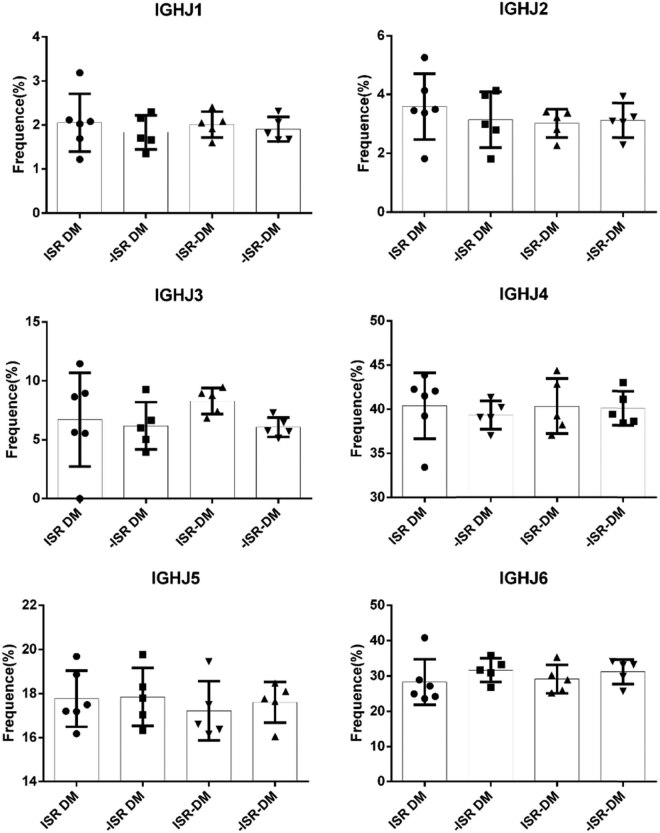
J gene usage of clonotypes in the four groups patients. Data show the percentage frequency of J gene by clonotypes in patients. Data show mean ± SD frequency of each individual. Each dot represents each individual patient’s information. Groups were compared using Student’s *t*-test. ISR: in-stent restenosis; DM: diabetes mellitus. IGHJ: immunoglobulin heavy joining.

**Figure A3:**
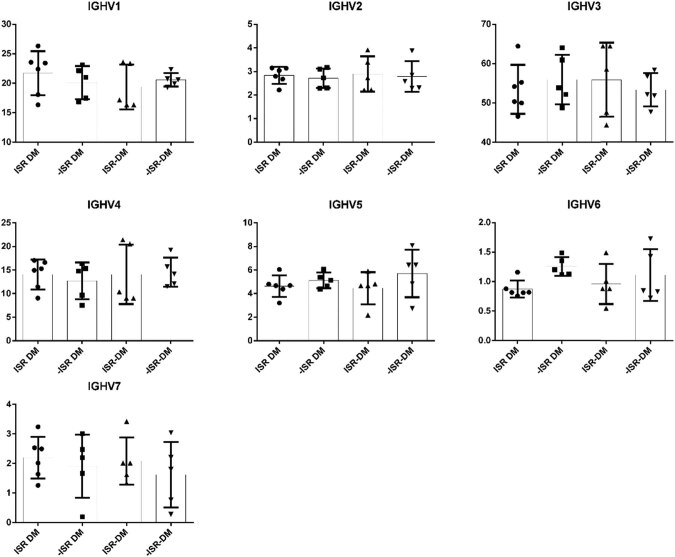
V gene usage of clonotypes in the four groups patients. Data show the percentage frequency of V gene by clonotypes in patients. Data show mean ± SD frequency of each individual. Each dot represents each individual patient’s information. Data were analyzed by using one-way ANOVA. ISR: in-stent restenosis; DM: diabetes mellitus.

IgH genes are assembled from a large pool of variable (V), delte (D), and joining (J) gene segments, and different V(D)J recombinations result in diversity of the BCR repertoire. In the present study, we did not find any significant differences in V gene or J gene segment usages within groups compared with between groups (Figures A2 and A3), with only some subdivisions of V gene segments showing differential usages (Figure 3). In DM patients, we found that four V subdivision genes exhibited differences with and without ISR, namely, IGHV1-18, IGHV1-3, IGHV2-70, and IGHV3-21 (Figure 3a). Meanwhile, IGHV3-30 was lower in ISR −DM patients than in −ISR DM patients, and a similar result was found in comparison with ISR DM patients (Figure 3b and c). Therefore, IGHV3-30 appears to be affected by both ISR and DM, but whether it is regulated by both ISR and DM in CAD patients requires further data.

The BCR repertoire is assembled by various numbers of V(D)J recombinations that affect the diversity. In total, 21 V/J paired gene usages differed between ISR DM and −ISR DM patients, comprising six downregulated genes and fifteen upregulated genes (Figure 3d). In non−DM patients, twelve V/J paired gene usages differed significantly in comparisons of patients with and without ISR (Figure 3e). Five upregulate V/J gene usages have differed significantly between ISR DM and ISR −DM (Figure 3f). Compared with the ISR DM group, the −ISR DM and ISR −DM groups both had significantly lower gene usages in V subdivision genes and V/J paired genes. These findings reveal that ISR and DM can produce changes in V/J paired gene usages and may have synergistic effects in CAD patients.

### 3.4 Receptor sharing between ISR and DM patients

**Figure 4:**
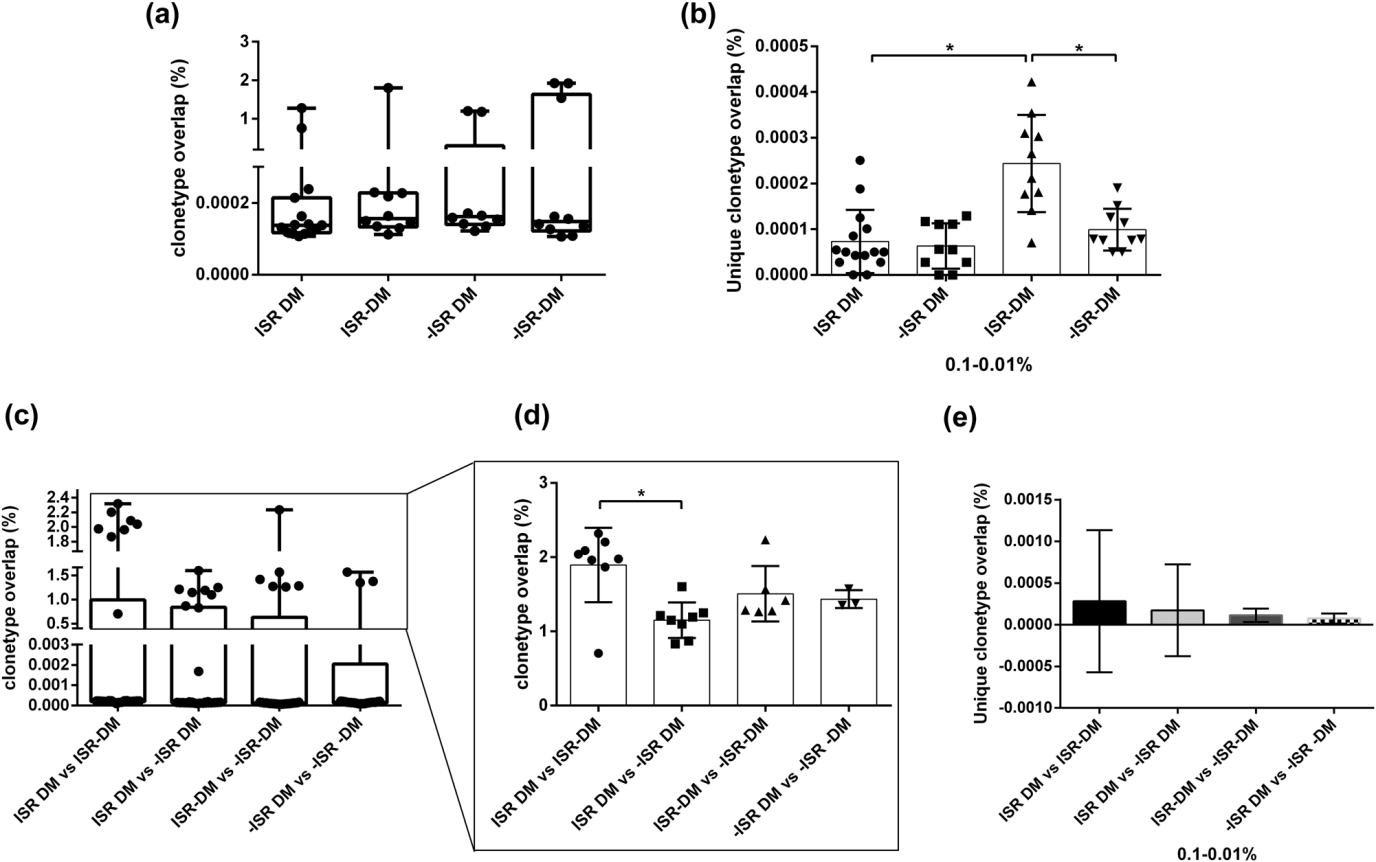
Unique clonotype overlap rate in each and between groups. (a) The data show the unique clonotype overlap of clonetype in ISR DM, ISR −DM, −ISR DM, −ISR −DM group. (b) The data showed the unique clonotype overlap between 0.1 and 0.01% in each group. (c) The data show the unique clonotype overlap rate between groups: ISR DM vs ISR −DM, ISR DM vs −ISR DM, ISR− DM vs −ISR −DM, −ISR DM vs −ISR −DM. (d) The data showed the unique clonotype overlap >0.1% between groups. (e) The data showed the unique clonotype overlap base of the sequence frequency between 0.1 and 0.01% within groups.

Next, we investigated the BCR sequences for shared sequences and determined whether sharing occurred between samples within groups. As shown in Figure 4a, there was no significant difference in the unique clonotype overlap rates in individual groups, but an increasing trend in overlaps was observed. The −ISR −DM group had the highest clonotype overlap rate, with an average overlap rate of 0.53% detected between any two −ISR, −DM patient samples, while ISR DM patients had the lowest overlap rate. Based on the section divisions described above, we analyzed the overlap rates based on the frequencies of the amino acid sequences. The results suggested that ISR and DM can change the shared amino acid sequences. We did not observe any differences in the section groups, except for the amino acid frequency between 0.1 and 0.01%. In the 0.1–0.01% section, ISR −DM patients had the highest rate among the four groups and differed significantly from the ISR DM and −ISR −DM groups (Figure 4b). Thus, it seems that ISR can increase the shared sequence rates, while DM can reverse this effect.

We further analyzed the clonotype overlap rates among the four groups. ISR DM and ISR −DM patients shared 0.50% clonotype overlaps (range: 0.001–2.31%). A similar result was found in the other parallels (Figure 4c). An interesting class polarization that can be observed in Figure 4c and d drew our attention. We collected the higher overlap rate classes and found differences for ISR DM versus ISR −DM and ISR DM versus −ISR DM. We also performed an analysis on the section groups described above, but found no significant difference in high or low frequencies of amino acid sequences (Figure 4e). Nevertheless, ISR and DM can alter the diversity by changing the shared sequences, and further investigations regarding BCR specificity and activation state are warranted.

## 4 Discussion

The development of coronary stents had led the field of interventional cardiology toward a new horizon. Although the introduction of balloon angioplasty and bare metal stent (BMS) implantation has improved interventional cardiology outcomes, there are approximately 20% overall with reported rates. The widespread use of coronary stents (BMS and DES) has enhanced our understanding and awareness of risk factors that may increase the incidence of restenosis, especially in DM patients for whom the risk of ISR can increase up to 30% [21,22,23]. The advent of DES implantation has further decreased the incidence of ISR. Despite the lower recurrence of ISR through advances in stent design and polymers, once restenosis has occurred in these stents, it becomes very challenging to treat and confers great suffering and heavy financial burdens on patients. In CAD patients, DM can accelerate atherosclerosis through infiltration of inflammatory cells (macrophages and T lymphocytes), larger necrotic core size, and more diffuse atherosclerosis [24].

During the progression of atherosclerosis, inflammatory pathways play important roles in formation of atherosclerotic plaques. A previous study suggested that inflammation can increase the occurrence of ISR, and that inflammatory biomarkers (IL-6, matrix metalloproteinases, C-reactive protein) have prognostic value in predicting the risk of ISR [25,26]. Among the infiltrating inflammatory cells, T lymphocytes have been extensively studied in atherosclerosis. Similar to Th1 cells that can secrete interferon-γ, T lymphocytes can activate macrophages and produce some molecules involved in plaque formation, such as Toll-like receptors (TLRs). Meanwhile, Th2 and Treg cells can secrete anti-inflammatory cytokines, such as IL-10 or transforming growth factor-β, which delay the occurrence of inflammation [24,27,28]. In our previous work, we found some differences of T cell receptor repertoires in CAD patients [29]. However, it has remained unclear how B cells contribute to plaque formation. In unpublished data, we found that B cells were involved in regulation of the immune state in CAD patients. Other reports revealed that B cells acted in a pro-inflammatory manner in CAD patients [7,8]. Cytokines produced by B cells can enhance immunomodulation during chronic inflammation; for example, TNF-α, IL-2, and IL-10 produced by B2 cells promoted atherosclerosis [7,10,11].

In DIO mice, B cells infiltrated the adipose tissue as an early response to DIO stimulation. However, the mechanism for the B cell function was not determined. Depletion of mature B2 cells by anti-BAFF antibodies resulted in proatherogenic chemokine production by macrophages [13]. B cell depletion was also a promising therapy for DM [30,31]. DM is a chronic inflammatory disease, in which elevation of pro-inflammatory molecules can induce cell surface TLRs and retinoic acid-inducible gene I (RIG-I)-like receptors, resulting in inflammation [32,33,34]. Furthermore, the signaling pathway transduction by TLRs and RIG-I was crucial for innate immunity and involved in both metabolic and cardiovascular diseases [35]. By throughput sequencing, we were able to monitor the immune state in DM patients. In a previous study, Seay et al. found that BCRs had a distinct tissue distribution and comparable diversity in DM patients [36]. In the present data, the B-cell clone diversity was changed by ISR and DM. Furthermore, DM affected the immune state mediated by B cells.

The present study had several limitations. First, our primary aim was to comprehensive analyze the profiling of BCR in ISR and DM patients. Because of the incidence rate of ISR, the study number was low and do not allow drawing solid conclusions. Second, the BCR diversity seems related to ISR or DM, but present data were insufficient to found any distinct amino acid characteristics associated with disease. These issues merit consideration when designing future study to confirm and expand the current findings.

## 5 Conclusion

In summary, we have performed a comprehensive characterization of the immune BCR profiles in CAD patients with ISR and DM. Based on the obtained sequence data, we found the ISR and DM can both change the diversity and clonal distribution of BCR repertoires in CAD patients. The V/J genes had different usages between ISR and DM patients. Furthermore, a polarization of shared sequences was found between groups and ISR −DM patients had the highest clonotype overlap rate among the groups. We also investigated the disease-associated clonotypes for sharing of common or distinct amino acid characteristics, and our determination of antigenic triggers responsible for these observations provides the potential for development of targeted therapies for ISR and DM patients.

## Appendix

**Table A1:** Sequenced read in patients with coronary in-stent restenosis

	#Sample_name	Total_seq_num	Merged_seq_num	Merged_seq_COPE	Merged_seq_java	Merged_seq_with_high_quality	Unmerged_seq	Merged_but_filter_by_low_quality	Unique_seq_num
ISR DM	A1	4692731.0 (100.00%)	4647538 (99.04%)	4456696 (94.97%)	190842 (4.07%)	4398031 (93.72%)	45193 (0.96%)	249507 (5.32%)	1720418 (36.66%)
	A2	3839975.0 (100.00%)	3802080 (99.01%)	3621462 (94.31%)	180618 (4.70%)	3600273 (93.76%)	37895 (0.99%)	201807 (5.26%)	1738505 (45.27%)
	A3	5057166.0 (100.00%)	5012295 (99.11%)	4806326 (95.04%)	205969 (4.07%)	4736013 (93.65%)	44871 (0.89%)	276282 (5.46%)	1892780 (37.43%)
	A4	5420795.0 (100.00%)	5372456 (99.11%)	5177788 (95.52%)	194668 (3.59%)	5095874 (94.01%)	48339 (0.89%)	276582 (5.10%)	1762116 (32.51%)
	A5	8001683.0 (100.00%)	7917514 (98.95%)	7703801 (96.28%)	213713 (2.67%)	7473555 (93.40%)	84169 (1.05%)	443959 (5.55%)	2660587 (33.25%)
	A6	3876484.0 (100.00%)	3838062 (99.01%)	3718589 (95.93%)	119473 (3.08%)	3637447 (93.83%)	38422 (0.99%)	200615 (5.18%)	1435631 (37.03%)
−ISR DM	B1	4386192.0 (100.00%)	4348027 (99.13%)	4174107 (95.16%)	173920 (3.97%)	4125054 (94.05%)	38165 (0.87%)	222973 (5.08%)	1735970 (39.58%)
	B2	5870338.0 (100.00%)	5817358 (99.10%)	5669411 (96.58%)	147947 (2.52%)	5481597 (93.38%)	52980 (0.90%)	335761 (5.72%)	2048152 (34.89%)
	B3	5416759.0 (100.00%)	5365685 (99.06%)	5217763 (96.33%)	147922 (2.73%)	5078140 (93.75%)	51074 (0.94%)	287545 (5.31%)	1671593 (30.86%)
	B4	4513400.0 (100.00%)	4477781 (99.21%)	4279651 (94.82%)	198130 (4.39%)	4260033 (94.39%)	35619 (0.79%)	217748 (4.82%)	1954041 (43.29%)
	B5	4518467.0 (100.00%)	4478493 (99.12%)	4274784 (94.61%)	203709 (4.51%)	4251282 (94.09%)	39974 (0.88%)	227211 (5.03%)	1651623 (36.55%)
ISR −DM	C1	4693943.0 (100.00%)	4654553 (99.16%)	4450592 (94.82%)	203961 (4.35%)	4414397 (94.04%)	39390 (0.84%)	240156 (5.12%)	1475989 (31.44%)
	C2	5228824.0 (100.00%)	5180855 (99.08%)	4968762 (95.03%)	212093 (4.06%)	4890450 (93.53%)	47969 (0.92%)	290405 (5.55%)	1628451 (31.14%)
	C3	4672150.0 (100.00%)	4632450 (99.15%)	4425797 (94.73%)	206653 (4.42%)	4392698 (94.02%)	39700 (0.85%)	239752 (5.13%)	1382597 (29.59%)
	C4	6004699.0 (100.00%)	5939467 (98.91%)	5764121 (95.99%)	175346 (2.92%)	5604723 (93.34%)	65232 (1.09%)	334744 (5.57%)	1790629 (29.82%)
	C5	4622544.0 (100.00%)	4576746 (99.01%)	4425203 (95.73%)	151543 (3.28%)	4342497 (93.94%)	45798 (0.99%)	234249 (5.07%)	1303608 (28.20%)
−ISR −DM	D1	6796199.0 (100.00%)	6731434 (99.05%)	6521023 (95.95%)	210411 (3.10%)	6371631 (93.75%)	64765 (0.95%)	359803 (5.29%)	2451801 (36.08%)
	D2	5348905.0 (100.00%)	5297828 (99.05%)	5135696 (96.01%)	162132 (3.03%)	4997924 (93.44%)	51077 (0.95%)	299904 (5.61%)	1693452 (31.66%)
	D3	5616682.0 (100.00%)	5563779 (99.06%)	5382779 (95.84%)	181000 (3.22%)	5261777 (93.68%)	52903 (0.94%)	302002 (5.38%)	2125012 (37.83%)
	D4	5165257.0 (100.00%)	5111731 (98.96%)	4943130 (95.70%)	168601 (3.26%)	4824062 (93.39%)	53526 (1.04%)	287669 (5.57%)	1966336 (38.07%)
	D5	4983236.0 (100.00%)	4935943 (99.05%)	4711896 (94.55%)	224047 (4.50%)	4692828 (94.17%)	47293 (0.95%)	243115 (4.88%)	1699089 (34.10%)

ISR: in-stent restenosis; DM: diabetes mellitus.
